# A Retrospective Case Series of Pericapsular Nerve Group (PENG) Block for Primary Versus Revision Total Hip Arthroplasty Analgesia

**DOI:** 10.7759/cureus.8200

**Published:** 2020-05-19

**Authors:** Promil Kukreja, Audrey Avila, Theresa Northern, Jyoti Dangle, Sree Kolli, Hari Kalagara

**Affiliations:** 1 Anesthesiology and Perioperative Medicine, University of Alabama at Birmingham, Birmingham, USA; 2 Anesthesiology, Cleveland Clinic, Cleveland, USA

**Keywords:** pericapsular nerve block, hip analgesia, peng, total hip arthroplasty, interfascial plane block, multimodal analesia

## Abstract

The innervation of the hip joint is complex and it is challenging to provide effective analgesia after hip surgery utilizing any single regional anesthesia technique. The pericapsular nerve group (PENG) block is an interfascial plane block aiming to block articular branches supplied by femoral, obturator, and accessory obturator nerves. In this case series, we compare the efficacy of the PENG block to provide analgesia in primary and revision total hip arthroplasty (THA). The ultrasound-guided PENG block technique is described and post-operative pain scores and opioid requirements are reported. The PENG block was successfully used in primary THA as a solo block, and it may be used in combination with other regional blocks for any hip surgery. The PENG block is an easy regional technique to perform in the supine position with motor-sparing benefits.

## Introduction

Total joint replacement is projected to become the most common elective surgical procedure in the coming decade; the prevalence of total hip arthroplasty (THA) was estimated as more than 2.5 million individuals in the entire United States population [1]. Osteoporosis and osteoarthritis contribute to the widespread necessity for hip surgery. An estimated two million fractures occur yearly in the United States [2]. THA is now the second most common joint replacement surgery. The number and rate of total hip replacements have increased greatly, doubling between 2000 and 2010 [3]. Orthopedic procedures involving the hip have remained challenging for regional anesthesia given the complex innervation, painful nature contributing to difficulty positioning, and a desire to maintain mobility to hasten postoperative recovery.

Chronic pain following THA is a complication that has been reported in 7%-28% of patients [4]. The effective postoperative analgesia is vital as acute surgical pain is a potential risk factor for future chronic pain. Persistent pain after THA (more than three months) is reported in 27% of patients and is reported to be correlated with the intensity of early postoperative pain rather than preoperative pain levels [5,6]. The majority of patients experience pain that is mild, especially when compared with their preoperative pain levels; however, 6% of patients report severe-extreme pain [7]. Multimodal anesthesia is paramount to limit the use of opioids and their association with adverse effects like respiratory depression, nausea and vomiting. Utilizing regional anesthesia helps to limit the use of opioids, however, which technique is best has yet to be determined. The lumbar plexus blocks, lumbar epidurals, and femoral nerve blocks have been associated with motor weakness. Fascia iliaca compartment block (FICB) has not been found to predictably decrease pain intensity or opioid use [8]. Quadratus lumborum (QL) block is a relatively new regional block found to provide effective analgesia after primary THA, but it can indirectly block lumbar plexus branches and may cause some motor weakness [9]. It is also a deep block and therefore contraindicated in patients on anticoagulation [10].

Recent anatomic studies by Short et al. confirmed the innervation of the anterior capsule to be the obturator nerve, accessory obturator nerve, and femoral nerve. These studies also evaluated the relationship with these nerves and other bony or soft tissue landmarks visible by ultrasound guidance [11]. Previous studies have found histologically that the anterior capsule has predominantly nociceptive fibers, while the posterior capsule is largely made up of mechanoreceptors [12]. The pericapsular nerve group (PENG) block was introduced to target and block these articular branches providing innervation to the hip. This regional anesthetic technique was described in 2018 by Giron-Arango et al. for acute analgesia related to hip fractures [13]. Given the case reports showing the efficacy of PENG blocks for hip fracture surgeries, we sought to investigate the analgesic efficacy of PENG blocks for primary and revision THAs. The PENG block targets only the sensory branches and not the posterior mechanoreceptors; there is a potential motor-sparing effect which is desirable for early ambulation, better physical therapy, and earlier discharge.

## Materials and methods

This retrospective case series included patients undergoing hip arthroplasty at a tertiary academic medical center. Six patients undergoing revision THA and six patients undergoing primary THA provided informed written consent for the nerve block. PENG block was performed preoperatively with the patient in a supine position. Two out of six patients for primary THA was done under spinal anesthesia and rest of the four under general endotracheal tube anesthesia (GETA), and all six out of six patients for revision THA were performed under GETA. The visual analog scores of pain in the post-anesthesia care unit (PACU) and at 6, 12, and 24 hours (hr) after surgery were obtained. In addition, cumulative oral morphine equivalent (OME) usage was obtained for PACU, for the first 6 hours, 6-12 hr, and 12-24 hr postoperatively.

Ultrasound-guided technique for PENG block

**Figure 1:**
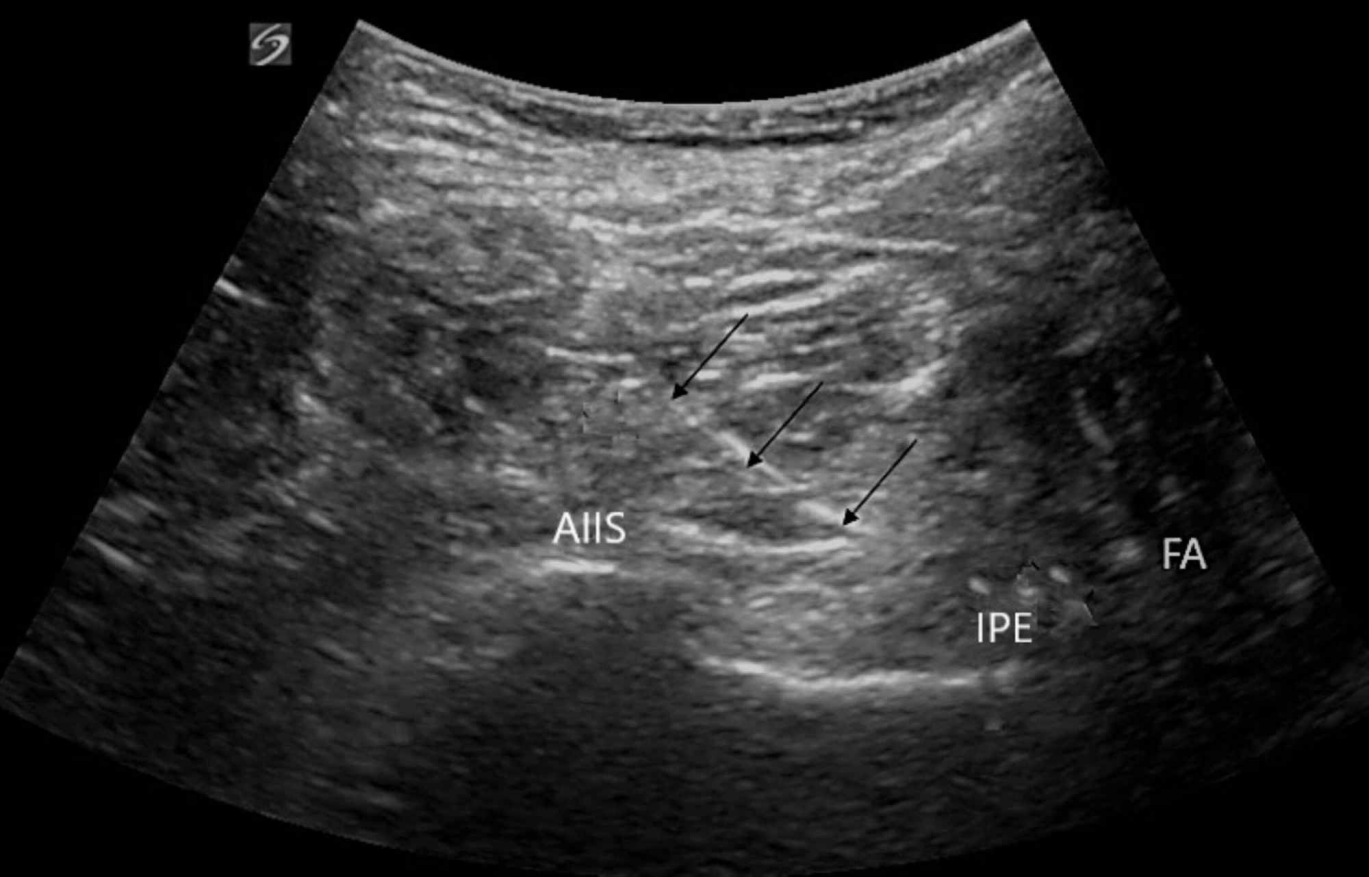
Ultrasound image of PENG block showing lateral to medial insertion of needle shown by black arrows passing the anterior inferior iliac spine (AIIS) and depositing the local anesthetic along the ilio-pubic eminence (IPE) lateral to the femoral artery (FA) PENG: pericapsular nerve group.

A low-frequency curvilinear transducer was placed in the transverse plane over the anterior inferior iliac spine (AIIS) and moved over inferiorly to visualize the pubic ramus. The femoral artery and ilio pubic eminence (IPE) were then visualized (Figure 1). Using in-plane technique 10 cm echogenic 21 gauge needle was advanced from lateral to medial direction, and 20 ml of local anesthetic 0.5% ropivacaine was deposited between the psoas tendon anteriorly and pubic ramus posteriorly.

## Results

In the primary THA group, average pain scores remained lower as compared to the revision THA group at all recorded time points during the study period (Tables 1-2).

**Table 1:** Post-operative pain scores for primary hip arthroplasty patients after the PENG block PACU: post-anesthesia care unit; PENG: pericapsular nerve group.

		Pain Scores			
Case	Age	PACU	6hrs	12hrs	24hrs
1	67	0	7	6	0
2	71	0	0	0	0
3	82	0	0	0	0
4	70	0	0	0	4
5	89	0	0	0	0
6	82	0	0	0	0

**Table 2:** Post-operative pain scores of revision hip arthroplasty patients after the PENG block PACU: post-anesthesia care unit; PENG: pericapsular nerve group.

		Pain Scores			
Case	Age	PACU	6hrs	12hrs	24hrs
1	75	8	0	0	0
2	67	0	8	0	0
3	67	9	7	0	2
4	60	0	2	0	0
5	59	6	4	10	3
6	62	8	5	0	3

**Figure 2:**
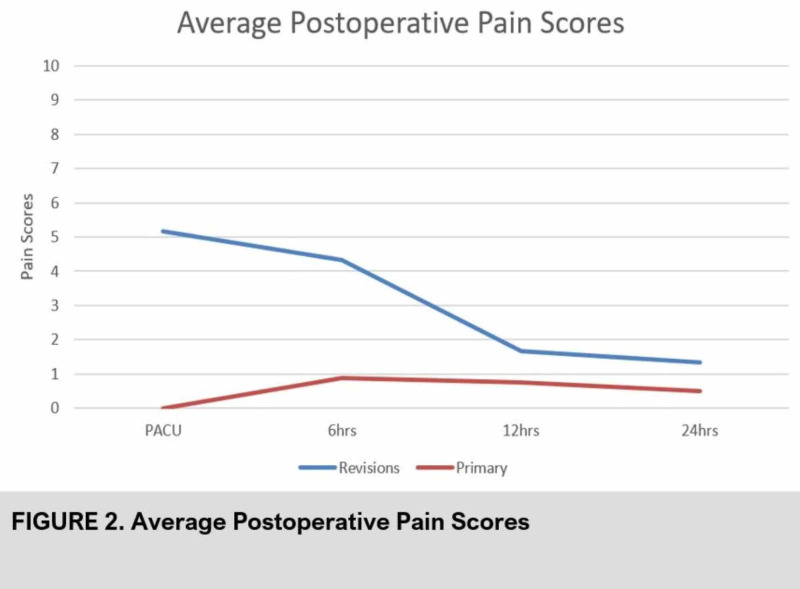
Average pain scores of primary versus revision hip arthroplasty patients after the pericapsular nerve group (PENG) block

In the revision group, average pain scores peaked in the PACU at 5.2 and decreased with time, as shown in Figure 2.

**Figure 3:**
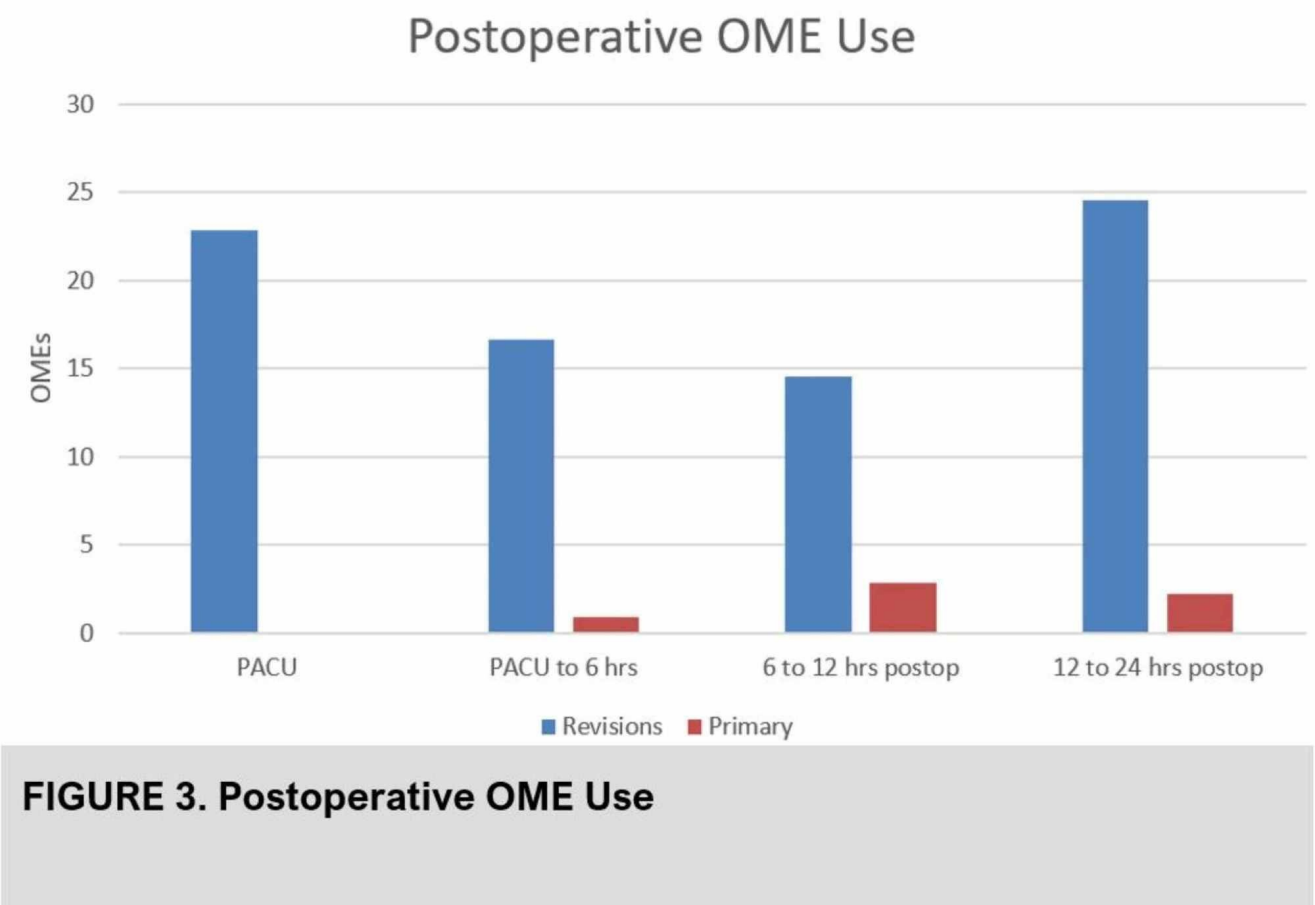
Post-operative opioid use (OME) of primary versus revision hip arthroplasty patients after pericapsular nerve group (PENG) block

Average postoperative opioid use in the first 24 hr was 78.7 OMEs in the revision group, compared to 18.4 OMEs in the primary group (Figure 3).

Five of the patients in the primary group had pain scores of 0 up until the 24 hour point and one patient in this group had 0 mg total OMEs in the first 24 hr.

## Discussion

The innervation of the hip joint is complex with the anterior capsule supplied predominantly with nociceptive fibers. The hip joint is a classic ball and socket joint formed by the acetabulum and the head of the femur. The hip joint is innervated by both lumbar (L1-L4) and sacral (L4-S4) plexus, receives its sensory innervation from the femoral, obturator, and sciatic nerves with contribution from nerve to quadratus femoris and superior gluteal nerve [14]. Articular branches of femoral, obturator, and accessory obturator nerves (AON) supply the hip joint. The PENG block is an interfascial plane block aiming to block these articular branches to enable hip analgesia.

Post-operative pain management after THA has always been a challenging goal to achieve. Multiple regional techniques have been used in the past, but there is no “best proven intervention” for THA analgesia [15]. The main regional techniques for THA include lumbar plexus block, lumbar epidural, femoral nerve block, sciatic nerve block, fascia iliaca block, pericapsular injection, or obturator nerve block. Unfortunately, all the above-mentioned blocks provide either inconsistent or partial analgesia, or are associated with lower extremity weakness that may interfere with physical therapy or increase the risk of a fall. Use of a lumbar epidural catheter or inadvertent epidural spread of lumbar plexus block can result in hypotension, leg weakness, and related adverse effects. The peripheral nerve blocks have been shown to be associated with falls after knee and hip arthroplasty [16]. A recent Cochrane review has demonstrated evidence supporting these blocks for a significant reduction in pain within 30 minutes of block placement [17]. Recently, a randomized controlled study concluded that anterior QL provided effective analgesia and decreased opioid requirements up to 48 hours after primary THA [9].

The coverage of articular nerve supply to the hip joint is critical for an effective analgesia. A small case series published initially along with the description of this novel PENG block showed good analgesic benefit for hip fractures. The median reduction of pain scores in this study was 7 points, showing a larger decrease in pain scores compared to other regional techniques in hip fractures [13]. The benefits of the PENG block are patient positioning for the procedure, no significant motor weakness, potential motor sparing effect, and analgesic efficacy [18]. The disadvantage is that it cannot be used as a sole anesthetic block for hip surgery and it can be used in combination with other nerve blocks like FICB for more extensive analgesia for hip surgery.

Postoperative pain scores and OME use were lower in the group undergoing primary hip surgery compared to revisions. One study of continuous femoral nerve blocks for THA showed opioid requirement of approximately 160 mg of OMEs and another study of continuous lumbar plexus block showed approximately 114 mg OMEs in first 48 hours [19]. In this case series, OME used in the primary THA group was significantly lower than the revision THA group in the first 24 hours. This reveals that PENG blocks may be a useful regional anesthetic technique for postoperative analgesia for primary hip surgery. Further randomized controlled trials with larger sample sizes are warranted to further elucidate the utility of PENG blocks for primary hip surgery and compare their effectiveness. PENG blocks in combination with lateral femoral cutaneous (LFCN) nerve block or local infiltration analgesia (LIA) may be needed for revision THA. Further studies are also needed to compare PENG blocks with QL blocks, fascia Iliaca blocks (FIB), suprainguinal fascia Iliaca block (SIFIB), and other combination of peripheral nerve blocks for hip arthroplasty analgesia.

There are some limitations to this case series such as small sample size, retrospective study design, and publication bias [20]. Also, we did not check sensory dermatomal levels to verify effective coverage from the PENG block. The surgical approach for the THA may affect the severity of post-operative pain. The complex innervation of hip and variation in anatomical planes where nerves run could explain the inconsistency of block results in revision THA [11]. At the same time, this case series explores a new approach for THA patients which can be utilized alone or in combination with other blocks to provide effective analgesia for hip surgery. Large sample size studies are warranted to further understand this new technique and also to compare its efficacy with traditional blocks for hip analgesia. Also, cadaveric and magnetic resonance imaging studies are required for a better understanding of the anatomical spread of local anesthetic and nerves covered with PENG block.

## Conclusions

The PENG block is an alternative regional analgesic technique for primary THA. It may be used as a potential supplement to other regional anesthesia techniques to effectively target articular branches to the hip joint. It can be used as an alternative to femoral nerve block or lumbar plexus block to prevent motor weakness. It can be performed easily in supine position using ultrasound guidance without any discomfort of patient positioning. The efficacy of the PENG block is not well established yet, randomized controlled trials are warranted for better understanding of PENG block with respect to analgesic efficacy and adverse effects.
